# miR-101 reverses hypomethylation of the PRDM16 promoter to disrupt mitochondrial function in astrocytoma cells

**DOI:** 10.18632/oncotarget.6652

**Published:** 2015-12-18

**Authors:** Qianqian Lei, Xiaoping Liu, Haijuan Fu, Yingnan Sun, Liping Wang, Gang Xu, Wei Wang, Zhibin Yu, Changhong Liu, Peiyao Li, Jianbo Feng, Guiyuan Li, Minghua Wu

**Affiliations:** ^1^ Hunan Provincial Tumor Hospital and The Affiliated Tumor Hospital of Xiangya Medical School, Central South University, Changsha 410013, Hunan, China; ^2^ Department of Breast Oncology, Sun Yat-Sen University Cancer Center, State Key Laboratory of Oncology in South China, Collaborative Innovation Center for Cancer Medicine, Guangzhou 510060, Guangdong, China; ^3^ Cancer Research Institute, School of Basic Medical Science, Central South University, Key Laboratory of Carcinogenesis and Cancer Invasion, Ministry of Education, Key Laboratory of Carcinogenesis, Ministry of Health, Changsha 410078, Hunan, China; ^4^ Department of Oncology, The First Hospital of Chenzhou City, 423000, Hunan, China

**Keywords:** hypomethylation, mitochondria, miR-101, ROS, PRDM16

## Abstract

Our previous report identified PR domain containing 16 (PRDM16), a member of the PR-domain gene family, as a new methylation associated gene in astrocytoma cells. This previous study also reported that miR-101 is a tumor suppressor in glioma. The present study confirms that PRDM16 is a hypomethylated gene that can be overexpressed in astrocytoma patients and demonstrates that the hypomethylation status of the PRDM16 promoter can predict poor prognoses for astrocytoma patients. The results reported herein show that PRDM16 was inhibited by miR-101 directly and also through epigenetic regulation. PRDM16 was confirmed as a new target of miR-101 and shown to be directly inhibited by miR-101. miR-101 also decreased the expression of PRDM16 by altering the methylation status of the PRDM16 promoter. miR-101 was associated with a decrease in the methylation-related histones H3K4me2 and H3K27me3 and an increase in H3K9me3 and H4K20me3 on the PRDM16 promoter. In addition, EZH2, EED and DNMT3A were identified as direct targets of miR-101, and miR-101 suppressed PRDM16 expression by targeting DNMT3A which decreases histone H3K27me3 and H3K4me2 at the PRDM16 core promoter. The results reported here demonstrate that miR-101 disrupted cellular mitochondrial function and induced cellular apoptosis via the mitochondrial pathway; for example, MMP and ATP levels decreased, while there was an increase in ADP/ATP ratios and ROS levels, levels of cleaved Caspase-9 and cleaved-PARP, the Bax/Bcl-2 ratios, and Smac release from the mitochondria to the cytoplasm. Knockdown of PRDM16 reversed the anti-apoptotic effect of miR-101 inhibition. In summary, miR-101 reversed the hypomethylation of the PRDM16 promoter which suppressed the expression of PRDM16, disrupted cellular mitochondrial function, and induced cellular apoptosis.

## INTRODUCTION

PRDM16 (PR domain containing 16) is a member of the PR-domain gene family. The PRDM family contains 17 members termed PRDM1 to PRDM17. The proteins encoded by these genes are a type of zinc finger transcription factor and contain N-terminal PR domains [1]. This protein family is involved in the transduction of signals that control cell proliferation, differentiation and apoptosis [2, 3, 4]. PRDM genes have important roles in human cancer, where they can act as tumor suppressors or oncogenes [5, 6]. Overexpression of PRDM2 induces cell apoptosis and differentiation, possibly via H3K9 histone methylation, in CML blast crisis cell lines [7, 8]. *PRDM14* is an oncogene that has increased expression in human lymphoid neoplasms [6]. PRDM16 has two protein isoforms, full-length PRDM16 and short isoform sPRDM16. These differ with regard to the presence or absence of the PR domain [9]. PRDM16 is also known as MEL1, which is highly expressed and significantly associated with poor prognoses in pediatric AML [10, 11]. In AML-NK patients, PRDM16 has a high level of expression due to promoter hypomethylation [12]. In a *Prdm16* knockout mouse model, the deletion of PDRM16 increased cell apoptosis [4]. However, studies regarding the PRDM family in glioma are less well understood. In a previous study, we determined the DNA methylome in gliomas using high-throughput methylated DNA IP combined with promoter and CpG island microarrays (MeDIP-Chip) [13]. The data indicated that the promoter of PRDM16 was hypomethylated. However, these results have yet to be validated.

Astrocytoma is the most common type of primary brain tumor and has a high incidence rate [14]. Due to its highly infiltrative and invasive nature, malignant astrocytoma has a dismal prognosis with a median survival period of approximately 12.1-14.6 months [15, 16]. Recurrence occurred in some patients because of the nonspecific targeting nature of current treatments [17]. Therefore, we urgently need to develop mechanistic-based approaches for astrocytoma management.

An increasing number of studies have shown the importance of miRNAs in carcinogenesis and their applicability as good targets for cancer therapy. miR-101 has been found to be expressed at low levels and to act as a tumor suppressor by targeting oncogenes in different type of cancers. miR-101 expression has been associated with the clinical prognosis of cancer patients. For instance, miR-101 has been shown to effectively inhibit cell proliferation and migration and to promote apoptosis by targeting Kruppel-like Factor 6 or EZH2 in glioblastoma stem cells or esophageal cancer cells, respectively [18, 19]. miR-101 has also been shown to suppress proliferation and the stem-cell-like phenotype of endometrial cancer cells by targeting EZH2, MCL-1 and FOS [20]. Our studies have shown that miR-101 can induce cell apoptosis or senescence by direct or epigenetic regulation to decrease the high expression levels of hypomethylated LMO3 [21] or CPEB1 [22] in astrocytoma cells. In addition, we have found that miR-101 can induce mitochondrial edema and vacuolar degeneration [21]. This paper further demonstrates the effects of miR-101 on mitochondrial function.

We authenticatedd the oncogenetic role of the hypomethylated gene PRDM16 and its effect on mitochondrial function and cell apoptosis as regulated by miR-101 in astrocytoma cells.

## RESULTS

### PRDM16 is highly expressed when its promoter is hypomethylated and this is correlated with poor outcomes in astrocytoma patients

**Figure 1:**
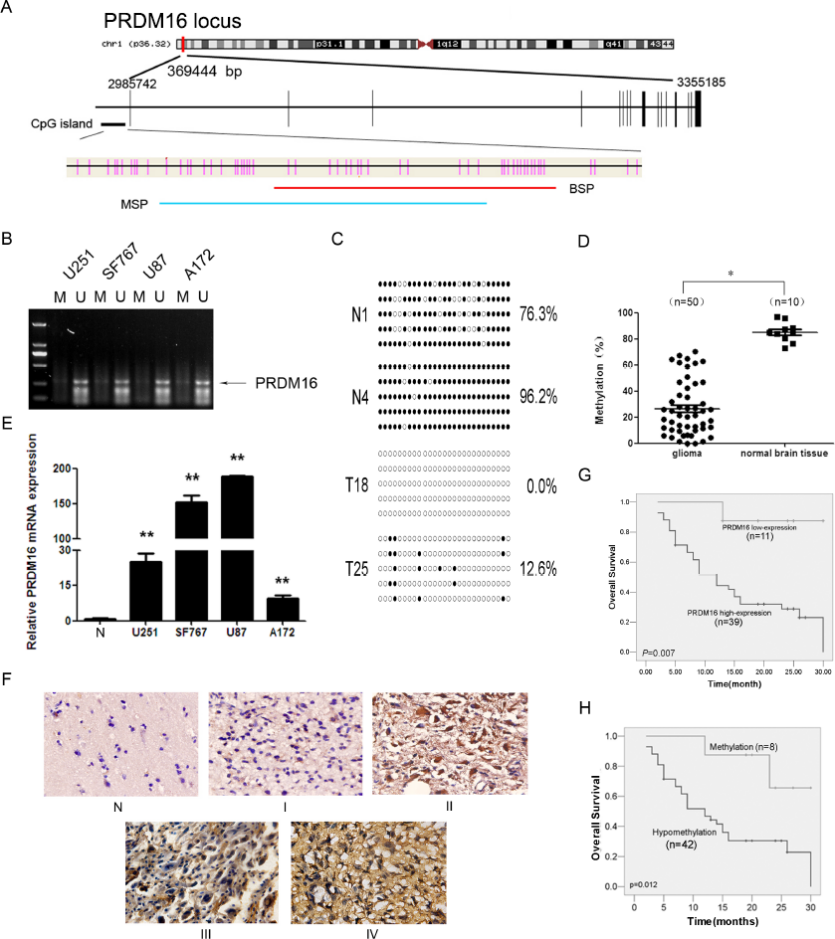
PRDM16 with a hypomethylated promoter is overexpressed and correlated with poor prognosis in astrocytoma patients **A.** A schematic diagram of the CpG dinucleotides within the PRDM16 promoter. The nucleotide number is relative to the transcription start site of PRDM16. The red line indicates the region that was tested with BSP; the blue line indicates the region that was detected with MSP. **B.** The methylation status of PRDM16 in the astrocytoma cell lines was detected using MSP. U, unmethylated primer; M, methylated primer. **C.** BSP of the upstream regulatory region of PRDM16 was performed for each representative tissue sample (N, normal brain tissue; T, glioma sample). For each sample, at least five separate clones were sequenced, and the results are shown here. Unmethylated CpG sites are shown as open circles, whereas methylated CpG sites are represented by closed circles. For each row of circles, the sequence results for an individual clone of the bisulfite-PCR product are given. The number of methylated CpGs divided by the total number of true CpGs analyzed is given as a percentage to the right of each BSP result. **D.** The methylation status of PRDM16 was detected using BSP in astrocytoma (n=50) and normal brain tissue samples (n=10), and the results were verified using an independent samples t-test. *P< 0.05. **E.** Real-time PCR was used to detect the expression of PRDM16. PRDM16 expression levels in normal brain tissue samples were much lower than in the four astrocytoma cell lines, and this was verified via an independent samples t-test. **P<0.01. **F.** PRDM16 expression levels in normal brain tissue and astrocytoma tissue samples were tested using ISH. **G.** The correlation between PRDM16 methylation in tumor tissues and the OS values of astrocytoma patients. Patients with PRDM16 hypomethylation had shorter OS values than patients with normal levels of PRDM16 methylation. For this analysis, the Kaplan-Meier method was used. **H.** The correlation between tumor PRDM16 protein expression levels and OS values for astrocytoma patients. Patients with high PRDM16 expression levels had poor outcomes. The Kaplan-Meier method was also used for this analysis.

BSP and MSP methods were used to further confirm the hypomethylation of PRDM16 in astrocytoma tumors (Figure 1A). We investigated the methylation status ofthe PRDM16 promoter in four astrocytoma cell lines using MSP. The unmethylated PRDM16 promoter was detectable, but the methylated PRDM16 promoter was nearly undetectable (Figure 1B). The BSP assay showed that the 27 CpG sites were heavily methylated in normal brain tissue samples, while only a few methylated CpG siteswere detected in the astrocytoma samples (Figure 1C). The levels of PRDM16 methylation in 50 astrocytoma tissues were lower than those in 10 normal brain tissuesamples (Figure 1D). No correlation was identified between sex, age and PRDM16 hypomethylation. A significant relationship between PRDM16 promoter hypomethylation and PRDM16 protein expression was observed. PRDM16 protein levels were amplified in astrocytoma cell lines (Figure 1E) and in 39 of 50 astrocytoma tissues. Comparison of methylation statuses and protein expression levels revealed that 36 of 39 tumors that had high PRDM16 expression levels exhibited hypomethylation. Levels of PRDM16 hypomethylation were also correlated to histological grades (P=0.047) (Table 1). PRDM16 expression was amplified in astrocytoma malignant grades (Figure 1F). Although the hypomethylation frequencies of PRDM16 in higher-grade astrocytomas (WHO grades III and IV) were higher than those in lower-grade astrocytomas (WHO grades I and II) (Table 1), no statistical correlation was found between PRDM16 expression and sex, age or histological grade (Table 2). For overall survival analysis, fifty astrocytoma patients were followed. The results revealed that lower PRDM16 expression or methylated PRDM16 correlated with higher survival rate, whereas PRDM16 hypomethylation or higher PRDM16 expression levels correlated with a lower survival rate (Figure 1G and 1H). These results suggest that high expression and hypomethylation of PRDM16 are involved in astrocytoma carcinogenesis and indicate poor prognoses for astrocytoma patients.

**Table 1:** Correlation between PRDM16 methylation status and clinical parameters of astrocytoma patients

Variable	PRDM16		P
	hypomethylation	methylation	
Total (N=50)	42	8	
Expression(score)			0.016
<8(11)	6	5	
≥8(39)	36	3	
Sex			0.377
Male(34)	30	4	
Female(16)	12	4	
Age(years)^a^			0.307
<42(23)	18	5	
>42(27)	24	3	
Grade			0.047
low grade (I+II)(20)	14	6	
high grade (II+III)(30)	28	2	

a median age is 42 years

Abbreviation: PRDM16, PR domain containing 16

**Table 2:** Relationship of PRDM16 expression and clinical parameters of astrocytoma patients

Variable	PRDM16		P
	<8 (score)	≥8 (score)	
Total (N=50)	11	39	
Sex			0.704
Male(34)	8	26	
Female(16)	3	13	
Age(years)^a^			0.468
<42(27)	7	20	
>42(23)	4	19	
Grade			0.382
low grade (I+II)(26)	7	19	
high grade (II+III)(24)	4	20	

a median age is 42 years

### Knockdown of PRDM16 disrupts mitochondrial function in U251 cells

**Figure 2:**
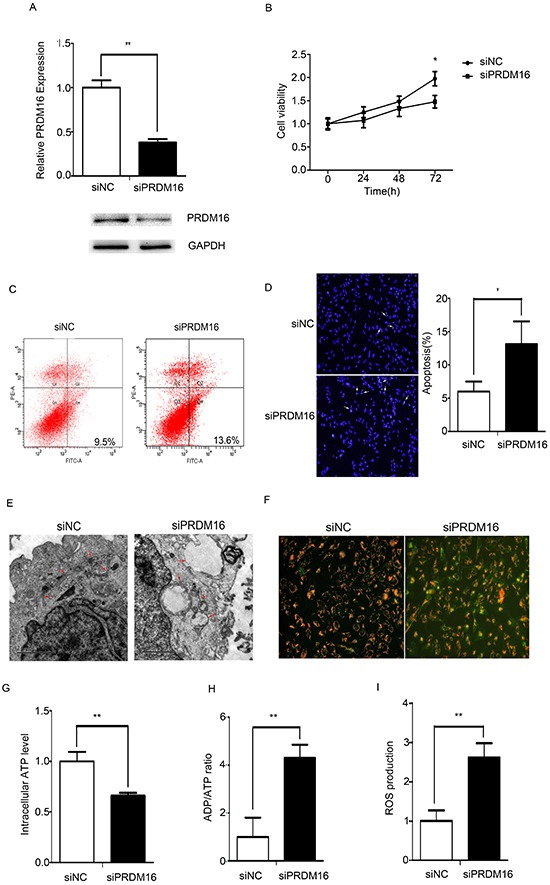
PRDM16 knockdown disrupted mitochondrial function in U251 cells **A.** Real-time PCR and western blot analyses were used to detect expression of endogenous PRDM16 after transfection with siRNA or control RNA. **B.** A cell growth assay was used to analyze the effects of PRDM16 siRNA on the proliferation of U251 cells. **C** and **D.** Flow cytometry and DAPI staining were performed to detect the effct of PRDM16 siRNA on cells apoptosis of U251 cells. **E.** Transmission electron micrograph was used to observe the effect o f PRDM16 siRNA on U251 cells morphologies. **F.** JC-1 staining analysis was used to detect mitochondrial membrane potentials after transfection with PRDM16 siRNA or control RNA. Red fluorescence indicates normal U251 cells, and green fluorescence indicates cells with mitochondrial dysfunction. **G, H.** and **I.** Mitochondrial ATP levels, ADP/ATP ratios and ROS production levels were detected 48 h after transfection with PRDM16 siRNA or control RNA.

Knockdown of endogenous PRDM16 in U251 cells was detected using real-time PCR and Western blots (Figure 2A). PRDM16 siRNA inhibited the proliferation of U251 cells (Figure 2B). Flow cytometry and DAPI staining were used to detect cell apoptosis. The knockdown of PRDM16 increased the apopototic frequency of the cells (Figure 2C and 2D). The morphologies of U251 cells transfected with PRDM16 siRNA were observed via transmission electron microscopy. The results revealed that PRDM16 siRNA treatment decreased the amount of mitochondria, and caused damage to the cristae structure and vacuolar degeneration (Figure 2E).

We wanted to determine whether PRDM16 siRNA disrupted mitochondrial function in U251 cells. The mitochondrial membrane potential indicated by the dye JC-1 is used to assess mitochondrial function [23]. As shown in Figure 2F, we found that the MMP was low and that JC-1 predominantly appeared as green fluorescence in U251 cells treated with PRDM16 siRNA. Mitochondrial dysfunction can inhibit ATP synthesis, increasing the cellular ADP/ATP ratio and ROS production [24]. In U251 cells treated with PRDM16 siRNA, ATP levels decreased (Figure 2G), and ADP/ATP ratios increased (Figure 2H). ROS levels increased when PRDM16 siRNA was transfected into U251 cells (Figure 2I).

### PRDM16 is a direct target gene of miR-101

**Figure 3:**
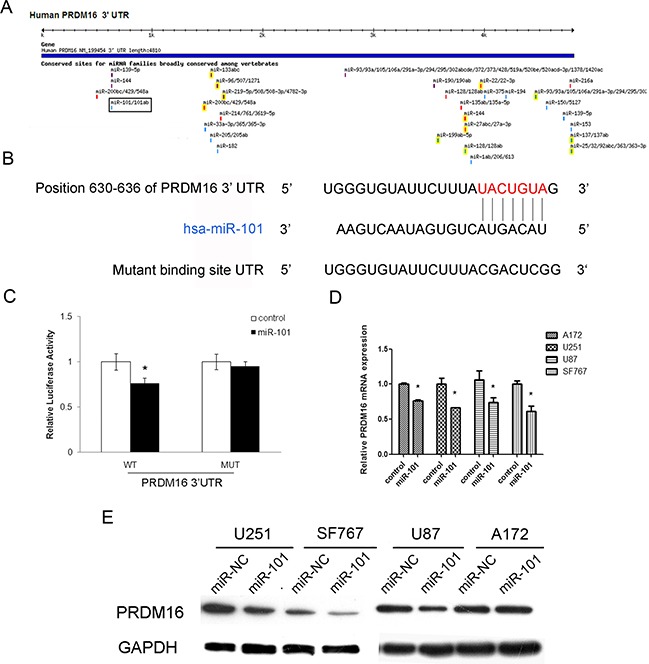
The PRDM16 gene is a direct target of miR-101 **A.** The PRDM16 gene was predicted to be a target of miR-101 using the online software program TargetScan 5.1. **B.** The miR-101 targeting site in PRDM16 (3′-UTR) is shown. Wild-type and mutated PRDM16 3′-UTRs are shown. **C.** miR-101 regulates the expression of PRDM16 3′-UTR reporter constructs. The luciferase reporter assays were performed 48 h after transfection with the indicated pMIR-REPORT plasmid and a Renilla transfection control plasmid, which were co-transfected with miR-101 or a scrambled control. The data shown are means ± S.D.s of six replicates and are representative of three independent experiments. An independent samples t-test was used. *P<0.05. **D.** miR-101 inhibits the expression of PRDM16 mRNA. Real-time PCR analysis was performed 48 h after transfection with miR-101 and a scrambled control. An independent samples t-test was used. *P<0.05. **E.** miR-101 regulates the expression of the PRDM16 protein in four astrocytoma cells. Western blot analysis was performed 72 h after transfection with miR-101 and a scrambled control. GAPDH was used as an internal control.

We used the online software TargetScan 5.1 (Cambridge, MA, USA) to predict potential miRNA-binding sites in the 3′-UTR sequence of PRDM16 and found that 3′-UTR of PRDM16 matched the seed sequence of miR-101, suggesting potential modulation of PRDM16 by miR-101 (Figure 3A). The predicted binding sites were cloned downstream of the firely luciferase gene in the pMIR-REPORT vector (Figure 3B). To verify whether PRDM16 is a direct target of miR-101, we constructed luciferase reporters with a wild-type (pMIR-PRDM16-3′-UTR-WT) or mutated 3′-UTR (pMIR-PRDM16-3′-UTR-Mut) of PRDM16. Both wild-type and mutated reporters were introduced into cells that were co-transfected with miR-101, and a significant decrease in luciferase activity was observed in wild-type compared with either mutant or empty vector controls co-transfected with miR-101 (Figure 3C). We used qRT-PCR and western blot techniques to examine the endogenous expression of PRDM16 in the presence of miR-101 in different astrocytoma cell lines. The expression of PRDM16 decreased at both the mRNA and protein levels in cells transfected with miR-101 mimics (Figures 3D and 3E). We concluded that the 3′-UTR of PRDM16 is directly targeted by miR-101 at the predicted binding site. Thus, PRDM16 may be considered a new target of miR-101 in astrocytoma cell lines.

### miR-101 reverses the hypomethylation of the PRDM16 promoter via histone methylation modification

**Figure 4:**
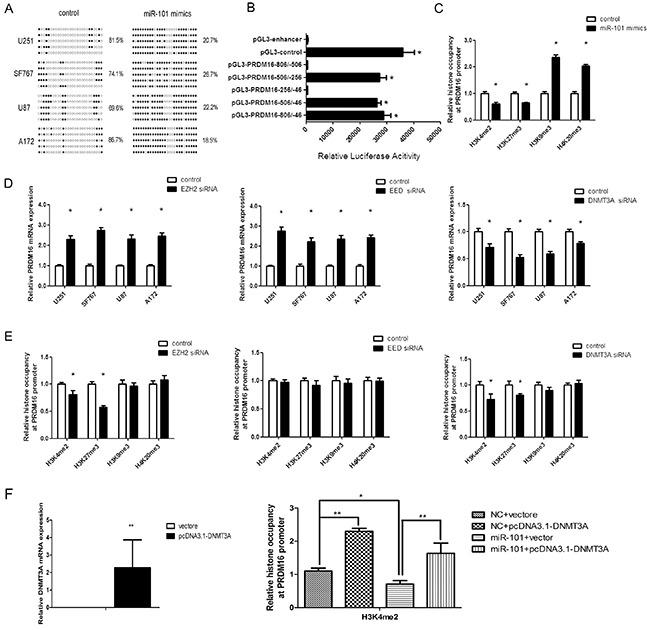
miR-101 reversed the hypomethylation status of the PRDM16 promoter **A.** PRDM16 methylation levels were increased by miR-101 in U251 cells. The unmethylated and methylated CpG sites are indicated by opened and closed circles, respectively. Each row indicates the sequencing result of one clone of the bisulfite-PCR product. The number of methylated CpGs was divided by the total number of true CpGs analyzed and is given as a percentage to the right of each BSP result. **B.** Analysis of promoter activity of the PRDM16 core promoter constructs via luciferase reporter assays. The construct containing the sequence spanning the region from −506 to −256 was sufficient to mediate maximal promoter activity. The core promoter ranged from −506 to −256. PGL3-control is the positive control, and pGL3-enhancer is the negative control. An independent samples t-test was used. *P<0.05. **C.** The histones occupancy of the PRDM16 promoter was affected by miR-101. A ChIP assay was used to detect the H3K4me2, H3K27me3, H3K9me3 and H4K20me3 occupancy the PRDM16 core promoter. U251 cells were transfected with miR-101 or a scrambled control. An independent samples t-test was used. *P<0.05. **D.** PRDM16 expression was regulated by EZH2, EED and DNMT3A. Real-time PCR analysis was performed 48 h after transfection with EZH2 siRNA, EED siRNA, DNMT3A siRNA or a scrambled control. An independent samples t-test was used. *P<0.05. **E.** The histones occupancy of the PRDM16 promoter was affected by EZH2 siRNA, EED siRNA and DNMT3A siRNA. A ChIP assay was performed to detect the H3K4me2, H3K27me3, H3K9me3 and H4K20me3 occupancy of the PRDM16 core promoter. U251 cells transfected with EZH2 siRNA, EED siRNA and DNMT3A siRNA were analyzed. An independent samples t-test was used. *P<0.05. **F.** Left: Real-time PCR was used to detect DNMT3A expression after transfection with a vector or pcDNA3.1-DNMT3A. Right: a ChIP assay was used to detect the H3K4me2 occupancy of the PRDM16 core promoter. U251 cells transfected with miR-101 and pcDNA3.1-DNMT3A were analyzed.

PRDM16, LMO3 and CPEB1 are hypomethylated genes in astrocytoma cells and because CPEB1 and LMO3 have been confirmed as epigenetic targets of miR-101[21], we examined the effects of miR-101 on the methylation status of PRDM16 by BSP. The methylation frequency of PRDM16 increased in astrocytoma cells transfected with miR-101 mimics, while miR-101 reversed the DNA hypomethylation levels of the PRDM16 promoter (Figure 4A).

Next, we tested the mechanism by which PRDM16 hypomethylation is reversed by miR-101. First, the luciferase reporter assay showed that the core promoter ranged from-506/-256 (Figure 4B). We examined H3K4me2, H3k9me3, H3K27me3 and H4K20me3 in chromatin associated with the PRDM16 promoter region using a ChIP assay. Both H3K4me2 and H3K27me3 were reduced at the PRDM16 core promoter in miR-101-treated astrocytoma cells compared with the control. However, H3K9me3 and H4K20me3 were increased (Figure 4C).

EZH2 and EED are components of PRC2, a complex associated with epigenetic modification linked to gene repression or activation and that can catalyze H3K27me3 methylation [21, 25]. Previous studies have also shown that EZH2, EED and DNMT3A are direct targets of miR-101 [26, 27, 28]. Therefore we examined the effects of EZH2, EED and DNMT3A on PRDM16 expression and associated histones at the PRDM16 core promoter. Knockdown of EZH2 or EED increased PRDM16 expression, whereas interfering with DNMT3A expression decreased PRDM16 expression (Figure 4D). Interestingly, knockdown of DNMT3A decreased the H3K27me3 and H3K4me2 at the PRDM16 promoter. Although knockdown of EZH2 decreased the H3K4me2 and H3K27me3 on the PRDM16 promoter, PRDM16 expression was not inhibited. Knockdown of EED did not influence histones on the PRDM16 promoter (Figure 4E). However, the levels of H3K4me2 and H3K27me3 on the PRDM16 promoter changed upon simultaneous treatment with miR-101 and DNMT3A (Figure 4F). These data indicated that miR-101 suppressed PRDM16 expression via DNMT3A-mediated modifications of histone H3K27me3 and H3K4me2 at the PRDM16 core promoter, and these effects did not occur though EZH2 and EED.

### miR-101 promotes cells apoptosis via the mitochondrial pathway

**Figure 5:**
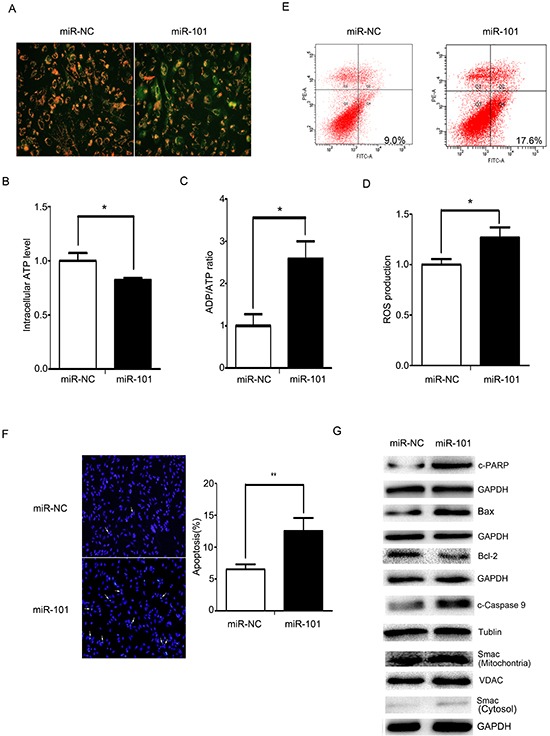
miR-101 induces cellular apoptosis via a mitochondrial pathway **A.** Mitochondrial membrane potentials were monitored by using JC-1 staining. U251 cells were transfected with miR-101 or a scrambled control. Red fluorescence indicates normal U251 cells, and green indicates cells with mitochondrial dysfunction. **B, C.** and **D.** Mitochondrial ATP levels, ADP/ATP ratios and ROS production were detected 48 h after transfection with miR-101 or a scrambled control. **E.** Flow cytometry analysis was performed 48 h after U251 cells transfection with miR-101 or a scrambled control. **F.** DAPI staining analysis was performed 48 h after U251 cells were transfected with miR-101 or a scrambled control. **G.** Western blot analysis of Bax, Bcl-2, caspase-9, c-PARP and Smac expression levels after transfection with miR-101 or a scrambled control. GAPDH and VDAC were used as loading controls for cytoplasmic protein and mitochondrial protein measurements, respectively.

As shown in Figure 5A, MMP decreased and JC-1 predominantly appeared as green fluorescence in U251 cells treated with miR-101 mimics. At the same time, ATP levels decreased in U251 cells treated with miR-101, and ADP/ATP ratios and ROS levels increased (Figure 5B, 5C and 5D). These data demonstrated that miR-101 also disrupted the mitochondrial function of U251 cells.

Defective mitochondria can generate excessive amounts of ROS, and high levels of ROS can induce apoptosis [29]. Compared with the control, the apoptotic rate increased in the U251 cells treated with miR-101, whereas treatment with the miR-101 inhibitor decreased apoptosis in U251 cells (Figures 5E and 5F). We also observed molecular changes in the mitochondrial-related apoptotic pathway. miR-101 overexpression resulted in increased caspase-9 cleavage and cleaved PARP protein levels. An increased Bax/Bcl-2 ratio facilitated the release of Smac from the mitochondria into the cytosol. Knockdown of miR-101 induced a decrease in cleaved PARP, cleaved caspase-9 and the Bax/Bcl-2 ratio and inhibited the release of Smac from mitochondria into the cytosol (Figure 5G). These data collectively indicated that miR-101 disrupted mitochondrial functions and promoted apoptosis in U251 cells by triggering the mitochondrial apoptotic pathway.

### Knockdown of PRDM16 reverses the effect of the miR-101 inhibitor on mitochondrial function and cell apoptosis in astrocytoma cells

**Figure 6:**
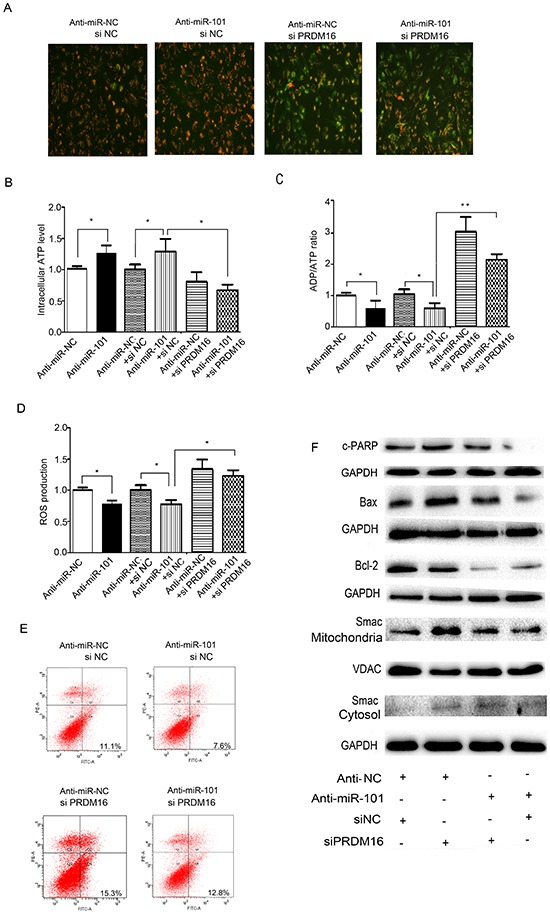
PRDM16 knockdown reversed the anti-apoptotic effect of miR-101 **A.** Mitochondrial membrane potentials were monitored by using JC-1 staining. U251 cells were transfected with anti-miR-NC or siNC, anti-miR-101 or siNC, anti-miR-NC or siPRDM16, and anti-miR-101 or siPRDM16, respectively. Red fluorescence indicates normal U251 cells, and green fluorescence indicates cells with mitochondrial dysfunction. **B, C and D.** Mitochondrial ATP levels, ADP/ATP ratios and ROS production levels were detected 48 h after transfection with anti-miR-NC, anti-miR-101, anti-miR-NC or siNC, anti-miR-101 or siNC, anti-miR-NC or siPRDM16, and anti-miR-101 or siPRDM16, respectively. **E.** Flow cytometry analysis was performed 48 h after transfection of U251 cells with anti-miR-NC or siNC, anti-miR-101 or siNC, anti-miR-NC or siPRDM16, and anti-miR-101 or siPRDM16, respectively. **F.** Western blot analysis of Bax, Bcl-2 and Smac expression levels after transfection with anti-miR-NC or siNC, anti-miR-101 or siNC, anti-miR-NC or siPRDM16, and anti-miR-101 or siPRDM16, respectively. GAPDH and VDAC were used as loading controls for cytoplasmic protein and mitochondrial protein levels, respectively.

Because we were not able to obtain the full length of the coding region of the PRDM16 gene, we were unable to detect the effects of PRDM16 overexpression on miR-101-induced cell apoptosis. We did, however, observe the effects of PRDM16 siRNA on the function of the miR-101 inhibitor. We found that the miR-101 inhibitor caused mitochondrial membrane potential increases in U251 cells. However, MMP levels were reduced when cells were treated with PRDM16 siRNA, and PRDM16 siRNA treatment reversed the increase in MMP levels caused by the miR-101 inhibitor. JC-1 mainly appeared as green fluorescence in U251 cells co-transfected with the miR-101 inhibitor and PRDM16 siRNA (Figure 6A). In addition, the miR-101 inhibitor increased ATP levels and decreased ADP/ATP ratios and ROS levels. Knockdown of PRDM16 reversed this increase in ATP levels, as well as the decreased ADP/ATP ratios and ROS production induced by the miR-101 inhibitor in U251 cells (Figure 6B, 6C and 6D). We found that the miR-101 inhibitor blocked cell apoptosis but cell apoptotic rates could be reversed by PRDM16 knockdown (Figure 6E). Knockdown of PRDM16 disrupted the suppression of cleaved PARP, cleaved caspase 9, the change to the Bax/Bcl-2 ratio, and the release of Smac from mitochondria to the cytoplasm caused by the miR-101 inhibitor in U251 cells (Figure 6F). Together, our results suggest that PRDM16 knockdown abrogated miR-101 inhibitor affected cell apoptosis and, therefore PRDM16 was involved in miR-101-induced cell apoptosis.

## DISCUSSION

Aberrant DNA methylation plays a crucial role in carcinogenesis [30]. Global loss of DNA methylation is one of the most common DNA methylome alterations in human cancers [31]. Hyper- or hypomethylation provide alternative mechanisms that can result in abnormal gene expression [32]. Many genes are silenced via hypermethylation of CpG islands in promoters [33–39]. Recently, researchers have focused on the hypomethylation of single copy genes and have found that these genes are activated in tumors [40–41]. Our data have verified four new hypomethylated genes, F10 [42], POTEH [43], LMO3 [21] and CPEB1 [22], and their prognostic values in astrocytomas. In this study, we confirmed that PRDM16 is a new hypomethylated gene in astrocytoma cells and we found that hypomethylation is one of the mechanisms responsible for high PRDM16 expression levels. Patients exhibiting high PRDM16 expression or promoter hypomethylation have poor chances of survival. Therefore PRDM16 can be considered a potential marker for the prognosis of astrocytoma patients. In addition to hypomethylation, miRNA dysregulation is also one of the causes of PRDM16 overexpression in astrocytoma cells. We discovered that miR-101 inhibited the expression of PRDM16 by directly binding to the 3′-UTR of PRDM16. PRDM16 was identified as a new target gene of miR-101.

Studies have identified methylation of K4, K9, K27, K36, and K79 on histone H3 and K20 on histone H4 [44]. H3K4 methylation is known to mark an open chromatin structure associated with active gene expression. Methylation of H3K9, H3K27 and H4K20 usually serve as markers for repressed gene expression. However, other studies have reported that H3K27 and H3K9 also increase gene expression [22, 25]. Our data show that miR-101 decreased the expression of PRDM16 by reversing the methylation status of the PRDM16 promoter. The methylation status was reversed by miR-101-induced decrease of the methylation-related histones H3K4me2 and H3K27me3 and an increase of H3K9me3 and H4K20me3 at the PRDM16 promoter.

EZH2 and EED are two components of polycomb repressive complex 2 (PRC2), which drives H3K27 trimethylation [45–47]. We found that H3K4me2 and H3K27me3 levels were decreased, but PRDM16 expression did not appear to be decreased when EZH2 or EED were knocked down. DNMTs comprise DNA methyltransferases, including DNMT1, DNMT2 and DNMT3 (DNMT3A and DNMT3B) [48]. Knockdown of DNMT3A decreased the H3K27me3 at the LMO3 promoter [21]. DNMT3A is also a target of miR-101 [28]. Our results show that the H3K4me2 and H3K27me3 at the promoter of PRDM16 were decreased and the expression of PRDM16 was decreased when U251 cells were treated with DNMT3A siRNA. Thus, we concluded that miR-101 suppressed PRDM16 expression by reversing the promoter hypomethylation through targeting DNMT3A.

Mitochondria are cellular organelles that are able to generate energy to maintain many cellular processes [49]. Mitochondrial dysfunction can result from cellular apoptosis and can therefore be considered a marker for apoptosis. When mitochondrial dysfunction occurs, it induces a decrease in mitochondrial membrane potential, generates excessive amounts of ROS and ATP, and alters apoptotic proteins [50–54]. Our previous data have demonstrated that miR-101 overexpression can damage the mitochondrial structures within astrocytoma cells and promote cell senescence [21] and apoptosis [22]. In this study, the ectopic expression of miR-101 induced cellular apoptosis and disrupted the function of mitochondria within astrocytoma cells by direct or epigenetic regulation of PRDM16. When the apoptotic signals of astrocytoma cells were activated by miR-101, MMP decreased, ADP/ATP ratios elevated, ATP levels decreased, and ROS levels increased. Bax is a pro-apoptotic protein and Bcl-2 is an anti-apoptotic protein, and an increased Bax/Bcl-2 ratio has been associated with cell apoptosis and shown to trigger the release of Smac/Diablo from mitochondria, as well as produce a caspase activation cascade [55, 56]. miR-101 increased Bax expression, decreased Bcl-2 expression, and promoted the release of the Smac protein from the mitochondria into the cytoplasm.

**Figure 7:**
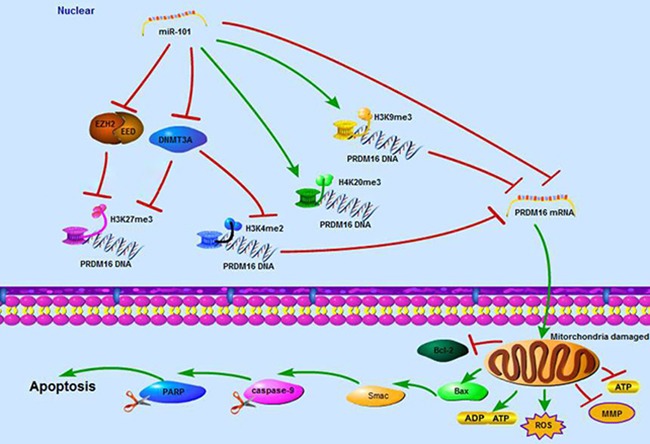
A schematic diagram of the working model for the miR-101 suppressing PRDM16 directly and epigenetically in astrocytoma cells

In summary, this study demonstrates that PRDM16 with a hypomethylated promoter has high expression levels correlated with poor prognoses of astrocytoma patients. The tumor suppressor miR-101 reverses the hypomethylation status of PRDM16 and suppresses the expression of PRDM16 through direct and epigenetic regulation. In addition, miR-101 disrupts cellular mitochondrial function and induces cellular apoptosis by inhibiting PRDM16 (Figure 7).

## MATERIALS AND METHODS

### Tissue specimens

We obtained frozen tissue samples of 50 gliomas and 10 normal brain tissues from the Xiangya Hospital of Central South University, Hunan, China, between January 2009 and July 2011. The study was approved by the Ethical Committee of the Faculty of Medicine, Central South University, and informed consent was obtained from all participating patients. Tumor samples were diagnosed using the World Health Organization system by two pathologists who were blinded to patient data. Clinical data, including gender, age, initial presentation, postoperative irradiation, chemotherapy, follow-up and outcomes, were obtained from medical records. The samples included 16 female and 34 male patients ranging from 16 to 65 years of age, with a mean age of 41 years old and a median age of 42 years old [21, 22].

### Cell lines and treatments

The Human astrocytoma cell lines U251, SF767, U87 and A172 were obtained from the Cell Center of Peking Union Medical College (Beijing, China). Cells were maintained in Dulbecco's Modified Eagle medium (Gibco, Grand Island, NY, USA) with 10% FBS, 100 units/ml of penicillin and 100 mg/ml of streptomycin at 37°C in a humidified atmosphere of 5% CO_2_ and 95% air.

### Genomic DNA isolation and bisulfite DNA treatment

Genomic DNA was isolated from each cell line and astrocytoma tissues and normal brain tissues were processed using the Universal Genomic DNA Extraction Kit Ver. 3.0 (Takara, Dalian, China) according to the manufacturer's instructions. Genomic DNA samples (0.5 mg) extracted from cells, tumors and normal tissue specimens were subjected to bisulfite treatment via an Epitect Bisulfite Kit (Qiagen, Hilden, Germany) and were subsequently stored at −20°C until further use.

### Bisulfite sequencing PCR (BSP) and methylation-specific PCR (MSP)

BSP and MSP studies were conducted as described previously [21, 22].

### miRNA and siRNA transfection

miR-101 mimics and their associated control, and miR-101 inhibitors and their associated control were synthesized by GenePharma Co., Ltd. (Shanghai, China). To generate a luciferase reporter construct, we synthesized 54 bp from the 3′-UTR of the PRDM16 mRNA from a human genomic DNA sample (Invitrogen). This 3′-UTR region of PRDM16, which contains the predicted target sites for miR-101, was then subcloned downstream of the pMIR-REPORT miRNA expression reporter vector (Ambion, Shanghai, China). We also constructed plasmids with mutated miR-101 target sites. MiR-101 mimics, miR-101 inhibitors and PRDM16 siRNA DNA samples were transfected using Lipofectamine 3000 (Life Technologies, Gaithersburg, MD, USA).

### Cloning of the PRDM16 promoter, plasmid construction and transfection

Different upstream regulatory regions of the PRDM16 gene were amplified from U251 DNA using PCR with UltraPF DNA polymerase (GeneCopoeia Inc., Rockville, MD, USA). The PCR fragments were digested with MluI/XhoI and linked to the luciferase-based promoter-less plasmid-pGL3-Enhancer Vector (Promega) to create the following plasmids: pGL3-PRDM16-806/-46, pGL3-PRDM16-506/-46, pGL3-PRDM16-256/-46, pGL3-PRDM16-506/-256 and pGL3-PRDM16-806/-506. The sequences and orientations of the cloned fragments were confirmed by direct DNA sequencing. The plasmids used for transfection were isolated and purified using a Purelink Plasmid Mini 25 Reaction Kit (Invitrogen—Life Technologies, Carlsbad, CA, USA). The promoter activities of these fragments were tested via transient transfection of 1 mg of plasmid DNA into the U251 cell lines using the Lipofectamine 3000. For the luciferase-based assay, the results were normalized against Renilla luciferase activity. At least three independent assays were performed.

### Luciferase reporter assay

U251 cells were plated in a 24-well plate and then cotransfected with 0.5 nmol of either miR-101 mimics or a scrambled control, 20 ng of either pMIR-PRDM16-3′-UTR-WT or pMIR-PRDM16-3′-UTR-MUT and 2 ng of pRL-TK (Promega). Cells were collected 48 h after transfection and analyzed using the Dual-Luciferase Reporter Assay System (Promega). Luciferase activity was detected using an M200 microplate fluorescence reader (Tecan, Beijing, China). The pMIR-REPORT-β-gal control vector was cotransfected as an internal control to correct for differences in both transfection and harvest efficiencies. Transfection experiments were performed in duplicate and were repeated in at least three independent experiments.

### Quantitative real-time PCR

Total RNA was extracted from glioma cells using TRIzol (Life Technologies, Rockville, MD, USA). For real-time PCR, 2 mg of the total RNA was reverse- transcribed using a cDNA synthesis kit (Fermentas, Burlington, ON, Canada). The β-actin gene was used as a control for this reaction. The data were normalized to β-actin levels, and levels of PRDM16 mRNA in the astrocytoma cell lines were determined using the 2^−ΔΔCt^ method. The miR-101 levels were determined using a SYBR-green-containing PCR kit (GenePharma Co.), and the RNA input was normalized to human U6 snRNA levels.

### Western blotting

The cell protein lysates, cytosol proteins and nuclear proteins were separated on 10% SDS-polyacrylamide gels, electrophoretically transferred to polyvinylidene difluoride membranes (Millipore, Danvers, MA, USA), and detected using rabbit polyclonal antibodies for PRDM16 (Santa Cruz Biotechnology, Santa Cruz County, CA, USA), mouse monoclonal antibodies for EZH2, EED (Santa Cruz Biotechnology) and GAPDH (Millipore), and rabbit polyclonal antibodies for H3K27me3, H3K9me3, H4K20me3 and H3K4me2 (Millipore) using a commercial ECL kit (Pierce, Rockford, IL, USA). The intensities of the protein fragments were quantified using ChemicalDocTM XRSþ (Bio-Rad, Berkeley, CA, USA).

### Annexin V assay

The apoptosis of U251 cells was quantified using a FITC-labeled AnnexinV/propidium iodide (PI) Apoptosis Detection kit (Beyotime, Beijing, China) according to the manufacturer's instructions. Flow cytometric analysis was performed immediately after supravital staining using a flow cytometer (Beckman, USA). Cells in early stages of apoptosis were AnnexinV positive; whereas cells that were both AnnexinV and PI positive were in the late stage of apoptosis.

### DAPI staining

The treated cells were first rinsed with PBS and then fixed with 4% paraformaldehyde in PBS for 1 h. Next, the cells were rinsed with PBS once again, permeabilized with 0.1% Triton X-100 for 2 min on ice, and washed with PBS twice. Cells were subsequently incubated with DAPI detecting liquid for 10 s at room temperature followed by microscopic observation and results were recorded.

### Mitochondrial membrane potential (MMP, Δψm)

To measure the mitochondrial membrane potential (Δψm), 5,5′,6,6′-tetrachloro-1,1′,3,3′-tetraethylbenzimidazolylcarbo-cyanine iodide (JC-1), which is a sensitive fluorescent probe for Δψm, was used (Beyotime, China). Treated or untreated cells were cultured in 24-well plates for 24 h, washed with PBS and incubated with a JC-1 working solution for 20 min at 37°C. The cells were rinsed twice with PBS, stained with 1 mL 10% DMEM medium containing 5 μmol/L JC-1, resuspended in 1 mL ice-cooled PBS, washed with PBS and resuspended in 500 μl PBS. The stained cells were analyzed using a fluorescence microscope to determine any changes in florescence from red to green.

### ATP activity experiment

ATP levels were measured by the luciferin-luciferase method following the protocol detailed in the ATP detection kit (Beyotime, China). After treatment, cells were collected and centrifuged at 1,000 g for 5 min. The pellets were treated with 200 μl lysis buffer from the ATP detection kit and were then centrifuged at 12,000 g for 5 min at 4°C. The supernatant was then transferred to a new tube for ATP testing. The luminescence from a 100-μl sample was assayed in a luminometer (Beckman, USA) together with 100 μl of ATP detection buffer from the ATP detection kit. A standard ATP concentration curve was prepared using known amounts of ATP (1nM—1mM).

### ADP/ATP ratio assay

The ADP/ATP assay was performed as instructed by Sigma. Adherent cells (10^3^–10^4^) could be cultured directly in the assay microplate. First, the culture medium was removed, after which 90 mL of ATP reagent was added to each well in the plate and the plate was tapped briefly to facilitate mixing. The plate was then incubated for 1 minute at room temperature. The luminescence (Beckman, USA) (relative light units) was measured using a luminometer for the ATP assay (RLU_A_). The plate was then incubated for an additional 10 minutes. After this 10-minute incubation, the luminescence was read and correlated to the associated ATP level (RLU_B_). This measurement provided the background prior to measuring ADP. Immediately following the RLUB reading, 5 μl of ADP reagent was added to each well, and the wells were mixed by tapping the plate or by pipetting. After 1 minute, the luminescence (RLU_C_) was read. The ADP/ATP ratio was calculated using the following formula: ADP/ATP ratio = (RLU_C_ – RLU_B_)/RLU_A._

### Measurements of reactive oxygen species

The production of reactive oxygen species (ROS) was measured using the ROS-sensitive dye carboxy-2, 7-dichlorodihydrofluorescein diacetate (H2DCFDA, Invitrogen) as an indicator. Briefly, glioma cells were homogenized in assay buffer, and the resulting homogenates were incubated with H2DCFDA at 37°C for 3 h. The fluorescent product formed was quantified using a spectrofluorometer that was set at 485/525 nm (Beckman, USA), and changes in fluorescence were expressed in arbitrary units.

### Immunohistochemical staining

Immunohistochemical studies were performed as described previously [21, 22].

### ChIP Assay and qRT-PCR

A total of 2×10^7^ cells of each parental U251 line were used for the ChIP assay. PCR-ChIP analysis was performed as previously described [21, 22].

### Follow-up study

The follow-up study was conducted as described previously [21, 22].

### Statistical analysis

Data are presented as means ± S.D.s obtained from at least three separate experiments. Multiple group comparisons were performed using ANOVA with a post hoc test for subsequent individual group comparisons. Differences in the PRDM16 promoter methylation statuses of normal brain tissues and astrocytoma tissues were examined using an independent samples t-test. The relationships between PRDM16 methylation status, protein expression and clinicopathological parameters were examined using the χ^2^-test. The OS curves were calculated using the Kaplan-Meier method, and the log-rank test was used to determine differences in OS rates between the two groups. The results were considered to be significant when a value of *P*<0.05 was obtained. All statistical analyses were performed using SPSS13.0 for Windows (SPSS Inc., Chicago, IL, USA).
